# Estimating the epidemic threshold on networks by deterministic connections

**DOI:** 10.1063/1.4901334

**Published:** 2014-11-12

**Authors:** Kezan Li, Xinchu Fu, Michael Small, Guanghu Zhu

**Affiliations:** 1 School of Mathematics and Computing Science, Guilin University of Electronic Technology, Guilin 541004, People's Republic of China; 2 Department of Mathematics, Shanghai University, Shanghai 200444, People's Republic of China; 3 School of Mathematics and Statistics, The University of Western Australia, Crawley, Western Australia 6009, Australia

## Abstract

For many epidemic networks some connections between nodes are treated as deterministic, while the remainder are random and have different connection probabilities. By applying spectral analysis to several constructed models, we find that one can estimate the epidemic thresholds of these networks by investigating information from only the deterministic connections. Nonetheless, in these models, generic nonuniform stochastic connections and heterogeneous community structure are also considered. The estimation of epidemic thresholds is achieved via inequalities with upper and lower bounds, which are found to be in very good agreement with numerical simulations. Since these deterministic connections are easier to detect than those stochastic connections, this work provides a feasible and effective method to estimate the epidemic thresholds in real epidemic networks.

In many real complex networks, it is well known that the connections between nodes are neither completely deterministic nor stochastic. In general, certain connections are deterministic, while the rest are random. For example, in a human epidemic network, each individual has generally deterministic connections with family, close friends, and relatives, while may have stochastic connections with colleagues, strangers, and so on. Moreover, information characterizing these deterministic connections can be more easily obtained than to adequately describe the behaviour of stochastic connections: that is, survey data and so on can provide an accurate picture of the deterministic, but not the stochastic contacts. When studying disease propagation, the epidemic threshold—the level of transmission at which a disease transitions from endemic to extinct—is the most important descriptor. Hence, when we study epidemic behavior on a complex network, it is extremely useful if we only need to use the deterministic connection information to estimate the corresponding epidemic threshold. In this paper, we apply theoretical analysis on several generic models and find that one can estimate the epidemic threshold from these deterministic connections and connection probability. Numerical simulations are further presented to both demonstrate and validate our results.

## I. INTRODUCTION

The analysis of the epidemic threshold is a very important topic for the study of the dynamical behavior and control methods for epidemic spreading on complex networks. Many reported results have shown that the topological structure of an epidemic network plays a vital role in its epidemic threshold. By using the heterogeneous mean-field (HMF) method on a standard SIS epidemic network,^1^ its epidemic threshold is given by $\beta_{c}=\langle k\rangle /\langle k^{2}\rangle$, where ⟨k⟩ and $\left\langle k^{2}\right\rangle$ are the first and second moments of the network degree distribution. By using linear stability analysis on an SIS Markovian epidemic network, a more exact result is that the epidemic threshold is given by *β_c_* = 1/*λ*_1_, where *λ*_1_ is the largest eigenvalue of the network's adjacency matrix.^2–4^ With this observation, the influence of the network topological characteristics on the spreading behaviour has been further investigated in depth.^5–8^ Finally, by embedding more realistic factors into traditional epidemic networks, many epidemic thresholds have been derived on multiplex networks such as epidemic networks with awareness,^9–13^ traffic-driven epidemic networks,^14,15^ epidemic networks with community structure,^16,17^ interconnected epidemic networks,^18–20^ time-varying epidemic networks,^21–23^ adaptive epidemic network,^24,25^ and so on.

While there is already much work addressing the epidemic thresholds of various epidemic networks, a thorough investigation of epidemic threshold of epidemic network with both deterministic and stochastic connections has not yet been done. In a real (social) epidemic network, each individual generally has both deterministic neighboring nodes (e.g., family and relatives) and stochastic nodes (e.g., colleagues and strangers). In fact, the well-known NW small-world network^26^ is generated using this same idea to address the transition properties between regular-lattice and random-lattice behavior in social networks. Consequently, the whole network can be divided into two unattached sub-networks: deterministic network and stochastic network. To the best of our knowledge, this network division method has not been applied to the study of epidemic transmission on networks or epidemic thresholds. In addition, different people have generally different probabilities for the connections with these stochastic neighboring nodes. Hence, the diversity of connection probability should be considered to obtain more reasonable models (see the case of nonuniform stochastic connections in Sec. IV). For universality, individual awareness and community structure will be also considered in our models. It is well known that many social networks have the community structures, where there exists a high density of intra-connections within each community, and a lower density of inter-connections between communities. In view of these (above) factors, to achieve the epidemic threshold estimation, we will first construct several SIS Markovian epidemic networks with deterministic and stochastic connections.

The main objective of this paper is to estimate the epidemic thresholds of these networks. In general, it is impossible to gain global connection information among all nodes in an epidemic network to calculate its exact epidemic threshold, as the network size is very large; the stochastic connections are time-varying, and so on. Therefore, it is natural to raise the following question: Can we estimate the epidemic thresholds of these networks using only the deterministic connection information?

In this paper, based on spectral analysis, we provide a positive answer to this question. By theoretical analysis, we have obtained some inequality estimations about the epidemic thresholds to give their upper bounds and lower bounds, which are just dependent on the topological structure of deterministic connections and stochastic connection probabilities. An optimal analysis for the upper bound is also developed. By using numerical simulations, these inequality estimations are shown to be extremely accurate.

The rest of this paper is organized as follows. In Sec. II, we give some preliminaries about epidemic network and graph theory. In Secs. III and IV, we estimate the thresholds of an epidemic network with uniform and nonuniform stochastic connection probability, respectively. In Sec. V, an epidemic network with community structure is considered. In Sec. VI, numerical simulations are given to verify the estimations in Secs. III–V. Finally, in Sec. VII, we conclude this paper.

## II. PRELIMINARIES

First, we provide some introductory remarks about complex networks and the spectral analysis of graphs.^27^ The topological structure of a complex network with size *n* can be represented by a graph *G*. The graph *G*, in turn, can be represented by its adjacency matrix *A* = (*a_ij_*)_*n*__×__*n*_, whose elements are either one or zero depending on whether there is a connection between nodes *i* and *j*. In this paper, we only consider undirected complex networks, i.e., the adjacency matrix is a real symmetric matrix. We say the pair of nodes (*i*, *j*) ∈ *G* means that the nodes *i* and *j* are connected, i.e., *a_ij_* = *a_ji_* = 1, otherwise, *a_ij_* = *a_ji_* = 0. It is assumed further that the graph *G* does not contain self loops (*a_ii_* = 0) nor multiple links between two nodes. The complement *G^c^* of the graph *G* consists of the same set of nodes but with (*i*, *j*) ∈ *G* if (*i*, *j*) ∉ *G^c^* and vice versa. The topological structure of *G^c^* is characterized by its adjacency matrix $A^{c}={\left(a_{ij}^{c}\right)}_{n\times n}$. According to graph theory, we can define that if *a_ij_* = 1, then $a_{ij}^{c}=0$; if *a_ij_* = 0, then $a_{ij}^{c}=1$; and $a_{ii}=a_{ii}^{c}=0$ for all *i* = 1,2,…, *n*. It is easy to see that *A^c^*^ ^= *J_n_* – *I_n_* – *A*, where *J_n_* is the all one matrix and *I_n_* is the identity matrix with order *n*.

Since the eigenvalues of the adjacency matrix *A* are real, they can be ordered as *λ*_1_(*A*) ≥ *λ*_2_(*A*) ≥⋯ ≥ *λ_n_*(*A*). The largest eigenvalue *λ*_1_(*A*) is also called the spectral radius of the graph. The largest and smallest eigenvalues often appear in the following supremum and infimum forms

$$\begin{array}{c} \lambda_{1}(A)=\underset{x\neq 0}{\sup}\frac{x^{T}Ax}{x^{T}x}=\underset{x^{T}x=1}{\sup}x^{T}Ax,\\ \lambda_{n}(A)=\underset{x\neq 0}{\inf}\frac{x^{T}Ax}{x^{T}x}=\underset{x^{T}x=1}{\inf}x^{T}Ax. \end{array}$$

*Lemma 1*. (Ref. 27) For symmetric *n* × *n* matrices *A*, *B*, it holds that

$$\lambda_{n}(B)+\lambda_{k}(A)\leq \lambda_{k}(A+B)\leq \lambda_{k}(A)+\lambda_{1}(B),$$ (1)

where *k* = 1,2,…, *n*.

Suppose that adjacency matrices $A=\left(\begin{array}{cc} A_{1} & 0\\ 0 & A_{2} \end{array}\right)$ and $B=\left(\begin{array}{cc} 0 & B_{12}\\ B_{12}^{T} & 0 \end{array}\right),A_{1}\in R^{m\times m},A_{2}\in R^{n\times n},B_{12}\in R^{m\times n}$. Obviously, the matrix $B_{12}B_{12}^{T}$ is semi-positive definite. From Ref. 27 (page 131), we know that if *λ* > 0 is an eigenvalue of $B_{12}B_{12}^{T}$, then $\pm \sqrt{\lambda }$ are two eigenvalues of *B*. So, by using Lemma 1, we have the following result.

*Lemma 2*. If *γ* ∈ [0, + *∞*), the largest eigenvalue of *A* + *γB* is bounded by

$$\begin{array}{c} \lambda_{1}(A+\gamma B)\leq \max\{\lambda_{1}(A_{1}),\lambda_{1}(A_{2})\}+\gamma \sqrt{\lambda_{1}(B_{12}B_{12}^{T})},\\ \lambda_{1}(A+\gamma B)\geq \max\{\lambda_{1}(A_{1}),\lambda_{1}(A_{2})\}-\gamma \sqrt{\lambda_{1}(B_{12}B_{12}^{T})}. \end{array}$$

*Lemma 3* (Perron-Frobenius Theorem^27^). An irreducible nonnegative *n* × n matrix A always has a real, positive eigenvalue λ_1_(A), and the modulus of any other eigenvalue does not exceed λ_1_(A). Moreover, λ_1_(A) is a simple zero of the characteristic polynomial det(A – λ*I_n_*). The eigenvector belonging to λ_1_(A) has positive components.

Now, we present the introduction about the traditional SIS Markovian epidemic network. In this network, each node can be in one of two distinct states at each time: susceptible (S) or infected (I). Each infected node can recover to be susceptible with probability *δ* in every time step. Each susceptible node has a probability *β* of contagion through contact with each of its infected neighbors. So, we can define an effective spreading rate *β*/*δ*. Without loss of generality, one can let *δ* = 1. Letting *p_i_*(*t*) denotes the probability of individual *i* to be infected at time *t* in the network, the dynamical process of epidemic spreading can be described by the following equations^12,13^ with continuous time:

$$\begin{array}{c} {\dot{p}}_{i}(t)=-p_{i}(t)+\beta (1-p_{i}(t))\sum_{j=1}^{n}a_{ij}p_{j}(t),\;i=1,2,\ldots ,n. \end{array}$$ (2)

In this case, all connections of the network are deterministic and characterized by adjacency matrix *A* = (*a_ij_*)_*n*__×__*n*_. By letting *p*(*t*) = (*p*_1_(*t*), *p*_2_(*t*),…, *p_n_*(*t*)), the Jacobian matrix at zero solution *p*(*t*) = 0 is given by −*I_n_* + *βA*. By the asymptotic stability condition,^2–4^ the zero solution is asymptotically stable if *λ*_1_(−*I_n_* + *βA*) < 0, which leads to the epidemic threshold *β_c_* = 1/*λ*_1_(*A*). If *β* is below *β_c_*, the infection will gradually die out, while if *β* is above *β_c_*, the infection spreads and becomes endemic.

In the following sections, we will consider the case where only some of the connections of the network are deterministic and the remainder is stochastic. Based on several mathematical models of the epidemic network, we focus on the study of estimating their epidemic thresholds by utilizing only the deterministic connection information.

## III. WITH UNIFORM STOCHASTIC CONNECTIONS

**FIG. 1.**
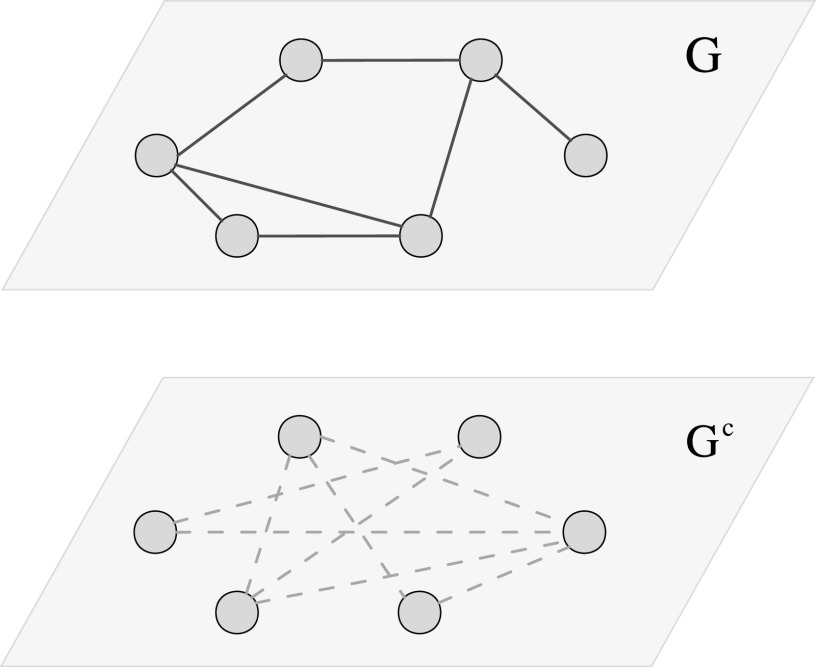
Example of an epidemic network with two unattached sub-networks: deterministic network *G* and stochastic network *G^c^*.

The whole network can be divided into two unattached sub-networks: deterministic network *G* and stochastic network *G^c^*. Fig. 1 presents a schematic diagram of an epidemic network with size *n* = 6. Any pair of nodes has deterministic connection in the deterministic network *G*, whose topological structure is characterized by an adjacency matrix *A* = (*a_ij_*)_*n*__×__*n*_, whose elements are either one or zero depending on whether there is a deterministic connection between nodes *i* and *j*. In addition, any pair of nodes has stochastic connection in the network *G^c^*, which is the complement of *G*. For each pair of nodes (*i*, *j*) ∈ *G^c^*, the connection probability between them is *α*, which means that the connections in stochastic network *G^c^* have uniform stochastic connections.

According to the above connection mechanism, the dynamical process of epidemic spreading can be described by the following equations:

$$\begin{array}{c} {\dot{p}}_{i}(t)=-p_{i}(t)+\beta (1-p_{i}(t))[\sum_{j=1}^{n}a_{ij}p_{j}(t)+\\ \alpha \sum_{j=1}^{n}a_{ij}^{c}p_{j}(t)],\;\;\;i=1,2,\ldots ,n. \end{array}$$ (3)

*Epidemic threshold and coupling matrix*—It is easy to get the Jacobian matrix at zero solution of network (3) as −*I_n_* + *βW*, where *W* = *A* + *αA^c^*. From the analysis in Sec. II, we know *β_c_* = 1/*λ*_1_(*W*). For convenience, we name matrix *W* as the coupling matrix in this paper. In fact, the coupling matrix *W* is a generalized form of adjacency matrix *A* in Sec. II. In order to estimate this epidemic threshold, we turn to seek the upper bound and lower bound of *λ*_1_(*W*) by only using adjacency matrix *A* and stochastic connection probability.

**Theorem 1**. Suppose *x* = (*x*_1_, *x*_2_,…, *x_n_*)^*T*^ ∈ *R^n^*, *Ax* = *λ*_1_(*A*)*x*, and *x^T^x* = 1. Then, the epidemic threshold of network (3) satisfies

$$\begin{array}{c} \beta_{c}\leq {\left[(1-\alpha )\lambda_{1}(A)+\alpha {\left(\sum_{i=1}^{n}x_{i}\right)}^{2}-\alpha \right]}^{-1},\\ \beta_{c}\geq {\left[(1-\alpha )\lambda_{1}(A)+\alpha n-\alpha \right]}^{-1}. \end{array}$$

*Proof*. Since for every *y* ∈ *R^n^*, *y^T^y* = 1,

$$\begin{array}{c} y^{T}Wy=y^{T}Ay+\alpha y^{T}A^{c}y\\ =y^{T}Ay+\alpha y^{T}(J_{n}-I_{n}-A)y\\ =(1-\alpha )y^{T}Ay+\alpha y^{T}J_{n}y-\alpha , \end{array}$$

we have

$$\begin{array}{c} \lambda_{1}(W)=\underset{y\in R^{n}}{\sup}y^{T}Wy\\ \leq (1-\alpha )\underset{y\in R^{n}}{\sup}y^{T}Ay+\alpha \underset{y\in R^{n}}{\sup}y^{T}J_{n}y-\alpha \\ =(1-\alpha )\lambda_{1}(A)+\alpha \lambda_{1}(J_{n})-\alpha \\ =(1-\alpha )\lambda_{1}(A)+\alpha n-\alpha . \end{array}$$ (4)

In addition, with *Ax* = *λ*_1_(*A*)*x*, we get

$$\begin{array}{c} \lambda_{1}(W)\geq x^{T}Wx=x^{T}Ax+\alpha x^{T}A^{c}x\\ =\lambda_{1}(A)+\alpha x^{T}(J_{n}-I_{n}-A)x\\ =(1-\alpha )\lambda_{1}(A)+\alpha {\left(\sum_{i=1}^{n}x_{i}\right)}^{2}-\alpha . \end{array}$$ (5)

By noting that *β_c_* = 1/*λ*_1_(*W*), we can obtain the inequalities in this theorem. *◻*

From Theorem 1, we can see that the upper and lower bounds of epidemic threshold *β_c_* only depend on the topological structure of graph *G* and connection probability *α*.

*Corollary 1*. If $\sum_{j=1}^{n}a_{ij}=k$ for all *i* = 1,2,…,*n*, then the epidemic threshold of network (3) is given by $\beta_{c}={\left[k+\alpha (n-k-1)\right]}^{-1}$.

*Proof*. If $\sum_{j=1}^{n}a_{ij}=k$ for all *i* = 1,2,…,*n*, we know that $x={\left(1/\sqrt{n},1/\sqrt{n},\ldots ,1/\sqrt{n}\right)}^{T}$ satisfies *Ax* = *λ*_1_(*A*)*x* = *kx*. From (5), we get $\lambda_{1}(W)\geq (1-\alpha )\lambda_{1}(A)+\alpha n-\alpha$. By combining Eqs. (4) and (5), we have $\lambda_{1}(W)=(1-\alpha )\lambda_{1}(A)+\alpha n-\alpha =k+\alpha (n-k-1)$, which leads to $\beta_{c}={\left[k+\alpha (n-k-1)\right]}^{-1}$. *◻*

For example, the NW small-world network with size *n* is generated with probability *α* for adding long-range connections, where each node is symmetrically connected with its *k* nearest neighbors in its initial nearest-neighbor network *G*. Obviously, $\sum_{j=1}^{n}a_{ij}=k$ for all *i* = 1,2,…,*n*. So, when we consider an epidemic dynamics in this network, from Corollary 1, its epidemic threshold is [*k* + *α*(*n* – *k* – 1)]^−1^, where *k* + *α*(*n* – *k* – 1) is the average degree of network *G*. This result is consistent with the theoretical threshold in homogenous epidemic network.^28^

## IV. NONUNIFORM STOCHASTIC CONNECTIONS

In general, due to the individual diversity, different nodes have different connection probabilities when they contact their neighboring stochastic nodes. That is to say, the spreading network generally includes nonuniform stochastic connections. To realize this connection mechanism, for (*i*, *j*) ∈ *G^c^*, let *d_ij_* be the probability with which the node *i* connects its stochastic neighbor node *j*. This means that if (*i*, *j*) ∈ *G^c^*, then there is a connection between them with probability *d_ij_d_ji_*. Certainly, if the stochastic transmission occurs only on some of the connections of *G^c^*, then the corresponding *d_ij_* = 0. In particular, in the case of uniform stochastic connections, we have *d_ij_d_ji_* = *α* for all (*i*, *j*) ∈ *G^c^*.

According to the above connection mechanism, the dynamical process of epidemic spreading can be described as

$$\begin{array}{c} {\dot{p}}_{i}(t)=-p_{i}(t)+\beta (1-p_{i}(t))[\sum_{j=1}^{n}a_{ij}p_{j}(t)+\\ \sum_{j=1}^{n}a_{ij}^{c}d_{ij}d_{ji}p_{j}(t)],\;\;\;i=1,2,\ldots ,n. \end{array}$$ (6)

The coupling matrix of network (6) can be written as

$$W=A+\sum_{(i,j)\in G^{c},i<j}D_{ij}A_{ij}^{c}D_{ij},$$ (7)

where

$$D_{ij}=\text{diag}\{0,\ldots ,0,\overset{i-\text{th}}{\sqrt{d_{ij}d_{ji}}},0,\ldots ,0,\overset{j-\text{th}}{\sqrt{d_{ij}d_{ji}}},0,\ldots ,0\},$$

and 0 – 1 symmetrical matrix

$$A_{ij}^{c}=\left(\begin{array}{cccccccc} 0 & & & \overset{i-\text{th}\;\text{col}}{0} & & \overset{j-\text{th}\;\text{col}}{0} & & 0\\ & \ddots & & \vdots & & \vdots & ⋰ & \\ & & & 0 & \cdots & 1 & & \\ & & & \vdots & & \vdots & & \\ & & & 1 & \cdots & 0 & & \\ & ⋰ & & \vdots & & \vdots & \ddots & \\ 0 & & & 0 & & 0 & & 0 \end{array}\right)$$

with only two displayed nonzero elements. It is easy to see that $\sum_{(i,j)\in G^{c},i<j}A_{ij}^{c}=A^{c}$. Let ○*G^c^*) be the number of connections in *G^c^*. Obviously, we have $○(G^{c})=\frac{n(n+1)}{2}-\sum_{1\leq i<j\leq n}a_{ij}$. Then, we attain the following theorem.

**Theorem 2**. Suppose *x* = (*x*_1_, *x*_2_,…, *x_n_*)^*T*^ ∈ *R^n^*, *Ax* = *λ*_1_(*A*)*x*, and *x^T^x* = 1. Then, the epidemic threshold of network (6) satisfies

$$\begin{array}{c} \beta_{c}\leq {\left[\lambda_{1}(A)+2\sum_{(i,j)\in G^{c},i<j}x_{i}x_{j}\right]}^{-1},\\ \beta_{c}\geq {\left[\lambda_{1}(A)+\frac{n(n+1)}{2}-\sum_{1\leq i<j\leq n}a_{ij}\right]}^{-1}. \end{array}$$

*Proof*. On one hand, for every *y* ∈ *R^n^*, *y^T^y* = 1, since $0\leq y_{i}^{2}+y_{j}^{2}\leq 1$ for all (*i*, *j*) ∈ *G^c^*, we get

$$\begin{array}{c} y^{T}Wy=y^{T}Ay+\sum_{(i,j)\in G^{c},i<j}y^{T}D_{ij}A_{ij}^{c}D_{ij}y\\ =y^{T}Ay+\sum_{(i,j)\in G^{c},i<j}d_{ij}d_{ji}(y_{i}^{2}+y_{j}^{2})\\ \leq \lambda_{1}(A)+\sum_{(i,j)\in G^{c},i<j}\left(y_{i}^{2}+y_{j}^{2}\right)\\ \leq \lambda_{1}(A)+○(G^{c})\\ =\lambda_{1}(A)+\frac{n(n+1)}{2}-\sum_{1\leq i<j\leq n}a_{ij}, \end{array}$$

which leads to

$$\begin{array}{c} \lambda_{1}(W)=\underset{y\in R^{n}}{\sup}y^{T}Wy\\ \leq \lambda_{1}(A)+\frac{n(n+1)}{2}-\sum_{1\leq i<j\leq n}a_{ij}. \end{array}$$ (8)

On the other hand, if *Ax* = *λ*_1_(*A*) *x* and *x^T^x* = 1, we have

$$\begin{array}{c} \lambda_{1}(W)\geq x^{T}Wx=x^{T}Ax+\sum_{(i,j)\in G^{c},i<j}x^{T}D_{ij}A_{ij}^{c}D_{ij}x\\ =\lambda_{1}(A)+\sum_{(i,j)\in G^{c},i<j}\left(x_{i}^{2}+x_{j}^{2}\right)\\ \geq \lambda_{1}(A)+2\sum_{(i,j)\in G^{c},i<j}x_{i}x_{j}. \end{array}$$ (9)

By noting that *β_c_* = 1/*λ*_1_(*W*), from (8) and (9), we can obtain the inequalities in this theorem. *◻*

As a special case, if *d_ij_* = *d_i_* for (*i*, *j*) ∈ *G^c^*, then the dynamical process of epidemic spreading can be described as

$$\begin{array}{c} {\dot{p}}_{i}(t)=-p_{i}(t)+\beta (1-p_{i}(t))[\sum_{j=1}^{n}a_{ij}p_{j}(t)\\ +\sum_{j=1}^{n}a_{ij}^{c}d_{i}d_{j}p_{j}(t)],\;\;\;i=1,2,\ldots ,n. \end{array}$$ (10)

The coupling matrix of network (10) is *W* = *A* + *DA^c^D*, where *D* = diag{*d*_1_, *d*_2_,…, *d_n_*}. Then, we obtain the following result.

**Theorem 3**. Suppose *x* = (*x*_1_, *x*_2_,…, *x_n_*)^*T*^ ∈ *R^n^*, *Ax* = *λ*_1_(*A*)*x,* and *x^T^x* = 1. Then, the epidemic threshold of network (10) satisfies

$$\begin{array}{c} \beta_{c}\leq {\left[\lambda_{1}(A)+\sum_{i,j=1}^{n}d_{i}d_{j}x_{i}x_{j}-(1+\lambda_{1}(A))\sum_{i=1}^{n}d_{i}^{2}x_{i}^{2}\right]}^{-1},\\ \beta_{c}\geq {\left[\lambda_{1}(A)+(n+\lambda_{1}(-A))\max\{d_{i}^{2}\}-\min\{d_{i}^{2}\}\right]}^{-1}. \end{array}$$

*Proof*. For every *y* ∈ *R^n^*, *y^T^y* = 1, since

$$y^{T}Wy=y^{T}Ay+y^{T}DJ_{n}Dy+y^{T}(-D^{2})y+y^{T}(-DAD)y,$$ (11)

we obtain

$$\begin{array}{c} \lambda_{1}(W)\leq \lambda_{1}(A)+\sup y^{T}DJ_{n}Dy\\ +\sup y^{T}(-D^{2})y+\sup y^{T}(-DAD)y. \end{array}$$ (12)

As $\frac{y^{T}DJ_{n}Dy}{{\left(Dy\right)}^{T}Dy}\leq \lambda_{1}(J_{n})$, we have $y^{T}DJ_{n}Dy\leq \lambda_{1}(J_{n}){\left(Dy\right)}^{T}Dy$, which leads to

$$\begin{array}{c} \sup y^{T}DJ_{n}Dy\leq \lambda_{1}(J_{n})\sup y^{T}(D^{2})y=\lambda_{1}(J_{n})\lambda_{1}(D^{2})\\ =n\max\{d_{i}^{2}\}. \end{array}$$ (13)

Similarly, we obtain

$$\begin{array}{c} \sup y^{T}(-DAD)y\leq \lambda_{1}(-A)\sup y^{T}(D^{2})y\\ =\lambda_{1}(-A)\max\{d_{i}^{2}\}. \end{array}$$ (14)

Obviously,

$$\sup y^{T}(-D^{2})y=\max\{-d_{i}^{2}\}=-\min\{d_{i}^{2}\}.$$ (15)

By integrating Eqs. (12)–(15), we conclude that

$$\lambda_{1}(W)\leq \lambda_{1}(A)+[n+\lambda_{1}(-A)]\max\{d_{i}^{2}\}-\min\{d_{i}^{2}\}.$$ (16)

From Eq. (11), if *Ax* = *λ*_1_(*A*)*x*, we have

$$\begin{array}{c} \lambda_{1}(W)\geq x^{T}Ax+x^{T}DJ_{n}Dx+x^{T}(-D^{2})x+x^{T}(-DAD)x\\ =\lambda_{1}(A)+\sum_{i,j=1}^{n}d_{i}d_{j}x_{i}x_{j}-\sum_{i=1}^{n}d_{i}^{2}x_{i}^{2}-x^{T}(DAD)x\\ \geq \lambda_{1}(A)+\sum_{i,j=1}^{n}d_{i}d_{j}x_{i}x_{j}-\sum_{i=1}^{n}d_{i}^{2}x_{i}^{2}-\lambda_{1}(A)x^{T}(D^{2})x\\ =\lambda_{1}(A)+\sum_{i,j=1}^{n}d_{i}d_{j}x_{i}x_{j}-[1+\lambda_{1}(A)]\sum_{i=1}^{n}d_{i}^{2}x_{i}^{2}. \end{array}$$ (17)

Therefore, by noting that *β_c_* = 1/*λ*_1_(*W*), we can obtain the result of this theorem. *◻*

Now, we give an application of Theorem 3 for an epidemic network with awareness. Suppose that all nodes in the network have individual protection awareness which is adjusted instantaneously by the infection density of their neighboring deterministic nodes. We find that the individual protection awareness will not change the epidemic threshold. For example, we consider the local protection awareness by letting connection probability

$${\bar{d}}_{i}(t)=d_{i}(1-\frac{1}{k_{i}}\sum_{j=1}^{n}a_{ij}p_{j}(t)),\;\;\;i=1,2,\ldots ,n.$$ (18)

With time-varying ${\bar{d}}_{i}$, network (10) can be rewritten as

$$\begin{array}{c} {\dot{p}}_{i}(t)=-p_{i}(t)+\beta (1-p_{i}(t))[\sum_{j=1}^{n}a_{ij}p_{j}(t)+\sum_{j=1}^{n}a_{ij}^{c}d_{i}d_{j}(1-\frac{1}{k_{i}}\sum_{l=1}^{n}a_{il}p_{l}(t))\\ \times (1-\frac{1}{k_{j}}\sum_{l=1}^{n}a_{jl}p_{l}(t))p_{j}(t)], \end{array}$$ (19)

where *i* = 1,2,…,*n*. It is obvious that the coupling matrix of network (19) is still *W* = *A* + *DA^c^D*. Thus, we have the following result.

*Corollary 2*. By embedding local protection awareness into network (10) with (18), its epidemic threshold remains constant. Moreover, the epidemic threshold estimation is also given by the inequalities in Theorem 3.

## V. UNIFORM STOCHASTIC CONNECTIONS AND COMMUNITY STRUCTURE

In this section, we consider that the deterministic network *G* has community structure. Without loss of generality, we suppose that *G* has two communities with sizes *m* and *n*, respectively. The inner connections within two communities are characterized by adjacency matrix $\left(\begin{array}{cc} A_{1} & 0\\ 0 & A_{2} \end{array}\right)$, where *A*_1_ ∈ *R^m^* and *A*_2_ ∈ *R^n^*. The outer connections between two communities are characterized by adjacency matrix $\left(\begin{array}{cc} 0 & B\\ B^{T} & 0 \end{array}\right)$, where *B* ∈ *R^m^*^ × *n*^. Then the adjacency matrix of deterministic network $G\;\mathrm{is}\;\text{A}=\left(\begin{array}{cc} A_{1} & 0\\ 0 & A_{2} \end{array}\right)+\left(\begin{array}{cc} 0 & B\\ B^{T} & 0 \end{array}\right)$, where *A*_1_ and *A*_2_ are symmetric, and *B* is generally asymmetric.

The dynamical process of epidemic spreading can be described by the following equations:

$$\begin{array}{c} {\dot{p}}_{i}(t)=-p_{i}(t)+\beta (1-p_{i}(t))[\sum_{j=1}^{m+n}a_{ij}p_{j}(t)+\alpha \sum_{j=1}^{m+n}a_{ij}^{c}p_{j}(t)],\;\;\;i=1,2,\ldots ,m+n. \end{array}$$ (20)

Define $B^{\dot{c}}=J_{n}^{m}-B$, where $J_{n}^{m}\in R^{m\times n}$ is the all one matrix. It is easy to verify that $B^{T\dot{c}}=B^{\dot{c}T}$. The coupling matrix of network (20) is

$$\begin{array}{c} W=A+\alpha A^{c}\\ =\left(\begin{array}{cc} A_{1}+\alpha A_{1}^{c} & 0\\ 0 & A_{2}+\alpha A_{2}^{c} \end{array}\right)+\left(\begin{array}{cc} 0 & B+\alpha B^{\dot{c}}\\ B^{T}+\alpha B^{T\dot{c}} & 0 \end{array}\right). \end{array}$$ (21)

**Theorem 4**. If *Az* = *λ*_1_(*A*)*z* and *z^T^z* = 1, the epidemic threshold of network (20) satisfies

$$\begin{array}{c} \beta_{c}\leq [(1-\alpha )\max\{\lambda_{1}(A_{1}),\lambda_{1}(A_{2})\}+\alpha {\left(\sum_{i=1}^{m+n}z_{i}\right)}^{2}\\ -(1-\alpha )\sqrt{\lambda_{1}(BB^{T})}-\alpha ]{\;}^{-1},\\ \beta_{c}\geq [(1-\alpha )\max\{\lambda_{1}(A_{1}),\lambda_{1}(A_{2})\}+\alpha (m+n)\\ +(1-\alpha )\sqrt{\lambda_{1}(BB^{T})}-\alpha ]{\;}^{-1}. \end{array}$$

*Proof*. From (4) and Lemma 2, we get

$$\begin{array}{c} \lambda_{1}(W)\leq (1-\alpha )\lambda_{1}(A)+\alpha (m+n)-\alpha \\ =(1-\alpha )\{\max(\lambda_{1}(A_{1}),\lambda_{1}(A_{2}))\\ +\sqrt{\lambda_{1}(BB^{T})}\}+\alpha (m+n)-\alpha . \end{array}$$ (22)

From (21) and (5), we get

$$\lambda_{1}(W)\geq (1-\alpha )\lambda_{1}(A)+\alpha {\left(\sum_{i=1}^{m+n}z_{i}\right)}^{2}-\alpha .$$

By applying Lemma 2, we have

$$\lambda_{1}(A)\geq \max\{\lambda_{1}(A_{1}),\lambda_{1}(A_{2})\}-\sqrt{\lambda_{1}(BB^{T})},$$

which results in

$$\begin{array}{c} \lambda_{1}(W)\geq (1-\alpha )\max\{\lambda_{1}(A_{1}),\lambda_{1}(A_{2})\}+\alpha {\left(\sum_{i=1}^{m+n}z_{i}\right)}^{2}\\ -(1-\alpha )\sqrt{\lambda_{1}(BB^{T})}-\alpha . \end{array}$$ (23)

So, by noting that *β_c_* = 1/*λ*_1_(*W*), we can obtain the result of this theorem. *◻*

Suppose *x* ∈ *R^m^*, *y* ∈ *R^n^*, *A*_1_*x* = *λ*_1_(*A*_1_)*x*, *A*_2_*y* = *λ*_1_(*A*_2_)*y,* and *x^T^x* = *y^T^y* = 1. Let $\mu_{1}=x^{T}By,\mu_{2}=x^{T}J_{m}x,\mu_{3}=y^{T}J_{n}y,\mu_{4}=x^{T}J_{n}^{m}y$. Let $\lambda_{L}=\max\{(1-\alpha )\lambda_{1}(A_{1})+\alpha \mu_{2}-\alpha ,(1-\alpha )\lambda_{1}(A_{2})+\alpha \mu_{3}-\alpha \}$, $s_{1}=(1/2){\left\{{\left\{(1-\alpha )[\lambda_{1}(A_{1})-\lambda_{1}(A_{2})]+\alpha (\mu_{2}-\mu_{3})\right\}}^{2}+{\left[(1-\alpha )\mu_{1}+\alpha \mu_{4}\right]}^{2}\right\}}^{\frac{1}{2}}$, and $s_{2}=(1/2)|(1-\alpha )[\lambda_{1}(A_{1})-\lambda_{1}(A_{2})]+\alpha (\mu_{2}-\mu_{3})|$. Now, to improve the estimation power, we present optimized upper bound for *β_c_* by using the Lagrange multipliers method.

*Corollary 3*. The optimal upper bound for *β_c_* of network (20) is given by ${\hat{\beta }}_{c}^{u}={\left(\lambda_{L}+s_{1}-s_{2}\right)}^{-1}$.

*Proof*. First, from Perron-Frobenius Theorem (see Lemma 3), we know that *x* > 0, *y* > 0, which leads to *μ*_1_ > 0, *μ*_2_ > 0, *μ*_3_ > 0, and *μ*_4_ > 0. Let *z* = (*ax^T^*, *by^T^*)^*T*^ with *a*^2 ^+ *b*^2 ^= 1, which means *z^T^z* = 1. From (21), we have

$$\begin{array}{c} z^{T}Wz={\left(\begin{array}{c} ax\\ by \end{array}\right)}^{T}\left(\begin{array}{cc} A_{1}+\alpha A_{1}^{c} & B+\alpha B^{\dot{c}}\\ B^{T}+\alpha B^{T\dot{c}} & A_{2}+\alpha A_{2}^{c} \end{array}\right)\left(\begin{array}{c} ax\\ by \end{array}\right)\\ ={\left(\begin{array}{c} ax\\ by \end{array}\right)}^{T}\left(\begin{array}{cc} A_{1} & B\\ B^{T} & A_{2} \end{array}\right)\left(\begin{array}{c} ax\\ by \end{array}\right)\\ +\alpha {\left(\begin{array}{c} ax\\ by \end{array}\right)}^{T}\left(\begin{array}{cc} A_{1}^{c} & B^{\dot{c}}\\ B^{T\dot{c}} & A_{2}^{c} \end{array}\right)\left(\begin{array}{c} ax\\ by \end{array}\right)\\ ={\left(\begin{array}{c} ax\\ by \end{array}\right)}^{T}\left(\begin{array}{cc} A_{1} & B\\ B^{T} & A_{2} \end{array}\right)\left(\begin{array}{c} ax\\ by \end{array}\right)\\ +\alpha {\left(\begin{array}{c} ax\\ by \end{array}\right)}^{T}\left(\begin{array}{cc} J_{m}-I_{m}-A_{1} & J_{n}^{m}-B\\ J_{m}^{n}-B^{T} & J_{m}-I_{m}-A_{2} \end{array}\right)\left(\begin{array}{c} ax\\ by \end{array}\right)\\ =a^{2}x^{T}A_{1}x+b^{2}y^{T}A_{2}y+2abx^{T}By\\ +\;\alpha (-a^{2}x^{T}A_{1}x-b^{2}y^{T}A_{2}y+a^{2}x^{T}J_{m}x\\ +\;b^{2}y^{T}J_{m}y-2abx^{T}By+2abx^{T}J_{n}^{m}y-a^{2}-b^{2})\\ =(1-\alpha )[a^{2}\lambda_{1}(A_{1})+b^{2}\lambda_{1}(A_{2})]+2(1-\alpha )ab\mu_{1}\\ +\;\alpha a^{2}\mu_{2}+\alpha b^{2}\mu_{3}+2\alpha ab\mu_{4}-\alpha . \end{array}$$ (24)

Let $f(a,b)=(1-\alpha )[a^{2}\lambda_{1}(A_{1})+b^{2}\lambda_{1}(A_{2})]+2(1-\alpha )ab\mu_{1}+\alpha a^{2}\mu_{2}+\alpha b^{2}\mu_{3}+2\alpha ab\mu_{4}-\alpha$. We need to solve the following optimization problem:

$$\{\begin{array}{cc} \max & f(a,b),\\ s.t. & a^{2}+b^{2}=1. \end{array}$$ (25)

If (a^*^, b^*^) is the optimal solution of (25), then ${\hat{\lambda }}_{L}=f(a^{*},b^{*})$ is the optimal lower bound for *λ*_1_(*W*), and ${\hat{\lambda }}_{L}^{-1}$ is the optimal upper bound for *β_c_*.

By the Lagrange multipliers method, we define the Lagrange function as

$$\begin{array}{c} L(a,b,\theta )=(1-\alpha )[a^{2}\lambda_{1}(A_{1})+b^{2}\lambda_{1}(A_{2})]\\ +\;2(1-\alpha )ab\mu_{1}+\alpha a^{2}\mu_{2}+\alpha b^{2}\mu_{3}\\ +\;2\alpha ab\mu_{4}-\alpha +\theta (a^{2}+b^{2}-1), \end{array}$$ (26)

where *θ* is the Lagrange multiplier. From the optimization condition $\frac{\partial L}{\partial a}=\frac{\partial L}{\partial b}=\frac{\partial L}{\partial \theta }=0$, we obtain

$$\begin{array}{c} 0=[(1-\alpha )\lambda_{1}(A_{1})+\alpha \mu_{2}]a+[(1-\alpha )\mu_{1}+\alpha \mu_{4}]b+\theta a,\\ 0=[(1-\alpha )\mu_{1}+\alpha \mu_{4}]a+[(1-\alpha )\lambda_{1}(A_{2})+\alpha \mu_{3}]b+\theta b,\\ 0=a^{2}+b^{2}-1. \end{array}$$ (27)

By adding the above first equation to the second equation, we have $\theta =-\alpha -{\hat{\lambda }}_{L}$. Since (*a*, *b*) ≠ (0, 0), we require that

$$\left|\begin{array}{cc} (1-\alpha )\lambda_{1}(A_{1})+\alpha \mu_{2}+\theta & (1-\alpha )\mu_{1}+\alpha \mu_{4}\\ (1-\alpha )\mu_{1}+\alpha \mu_{4} & (1-\alpha )\lambda_{1}(A_{2})+\alpha \mu_{3}+\theta \end{array}\right|=0.$$

From above equation, we obtain

$$\begin{array}{c} {\hat{\lambda }}_{L}=-\alpha -\theta \\ =\frac{\left(1-\alpha )[\lambda_{1}(A_{1})+\lambda_{1}(A_{2})]+\alpha (\mu_{2}+\mu_{3}\right)}{2}+s_{1}-\alpha \\ =\lambda_{L}+s_{1}-s_{2}. \end{array}$$ (28)

By combining (27) and (28), we have

$$\begin{array}{c} a^{*}=\frac{\pm [(1-\alpha )\mu_{1}+\alpha \mu_{4}]}{\sqrt{{\left[(1-\alpha )\lambda_{1}(A_{1})+\alpha \mu_{2}+\theta \right]}^{2}+{\left[(1-\alpha )\mu_{1}+\alpha \mu_{4}\right]}^{2}}},\\ b^{*}=\frac{\pm |(1-\alpha )\lambda_{1}(A_{1})+\alpha \mu_{2}+\theta |}{\sqrt{{\left[(1-\alpha )\lambda_{1}(A_{1})+\alpha \mu_{2}+\theta \right]}^{2}+{\left[(1-\alpha )\mu_{1}+\alpha \mu_{4}\right]}^{2}}}. \end{array}$$

Since *μ*_1_ > 0, *μ*_4_ > 0, from the definition of *f*(*a*, *b*), the optimal solution (a^*^, b^*^) of Eq. (25) should satisfy a^*^b^* ^> 0, i.e., they have the same sign. Finally, by noting *β_c_* = 1/*λ*_1_(*W*), equality (28) gives the result of this corollary. *◻*

## VI. NUMERICAL SIMULATIONS

In this section, we present some numerical examples to show the effectiveness of epidemic threshold estimations in Secs. III–V.

**FIG. 2.**
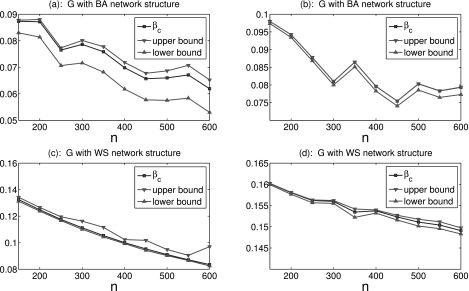
Comparisons between the epidemic threshold *β_c_* and upper-lower bound estimation under different network sizes *n* and uniform stochastic probability *α* = 0.01 in (a), (c), and *α* = 0.001 in (b), (d).

First, we consider the case of epidemic network with uniform stochastic connections. Without loss of generality, the topological structure of deterministic network *G* is characterized by WS small-world network^29^ or BA network.^30^ The WS network is generated with probability 0.1 for rewiring links, where each node is symmetrically connected with its six nearest neighbors in its initial nearest-neighbor network. The BA network is produced with four initial nodes, which are fully connected, and then adding a new node with three new edges at each time step. The epidemic threshold is computed by *β_c_* = 1/*λ*_1_(*W*). The upper bound and lower bound are given by Theorem 1. Fig. 2 gives some comparisons between the epidemic threshold and upper-lower bound estimation under different network size *n* and uniform stochastic probability *α*. From this figure, we can see that the epidemic threshold is always bounded by upper bound and lower bound.

**FIG. 3.**
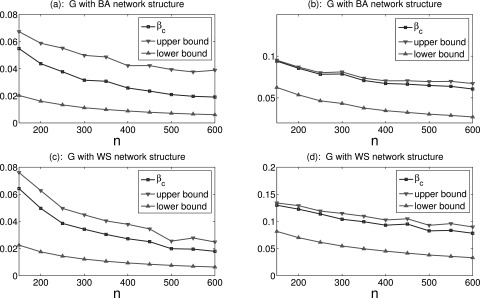
Comparisons between the epidemic threshold *β_c_* and upper-lower bound estimation under different network sizes *n* and parameter *η* = 0.5 in (a), (c), and *η* = 0.2 in (b), (d).

**FIG. 4.**
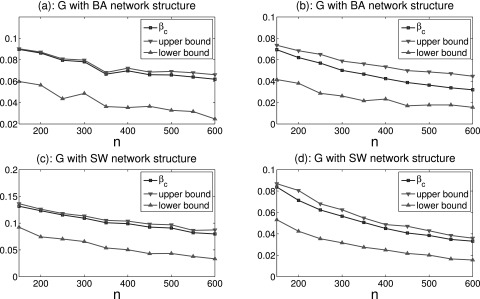
Comparisons between the epidemic threshold *β_c_* and upper-lower bound estimation under different network sizes *n* and parameters *μ* = 0.1, *σ*^2 ^= 0.001 in (a), (c), and *μ* = 0.2, *σ*^2 ^= 0.001 in (b), (d).

Next, we consider the case of epidemic network with nonuniform stochastic connections. Suppose that ${\left\{d_{i}\right\}}_{i=1}^{n}$ are uniformly distributed within [0, *η*] with 0 < *η* ≤ 1. Fig. 3 shows some comparisons between the epidemic threshold and upper-lower bound estimation under different network size *n* and parameter *η*. By decreasing parameter *η*, we can reduce statistically the number of stochastic connections. This figure verifies the upper-lower bound estimation in Theorem 3 very well. Integrating Figs. 2 and 3, it can be concluded that the smaller the stochastic connection probability is, the better the estimation will be. In order to explore the influence resulting from distribution, we further suppose that *d_i_* is generated from a normal distribution with mean *μ* and variance *σ*^2^. The result is presented by Fig. 4, in which the upper-lower bound estimation in Theorem 3 is still valid.

**FIG. 5.**
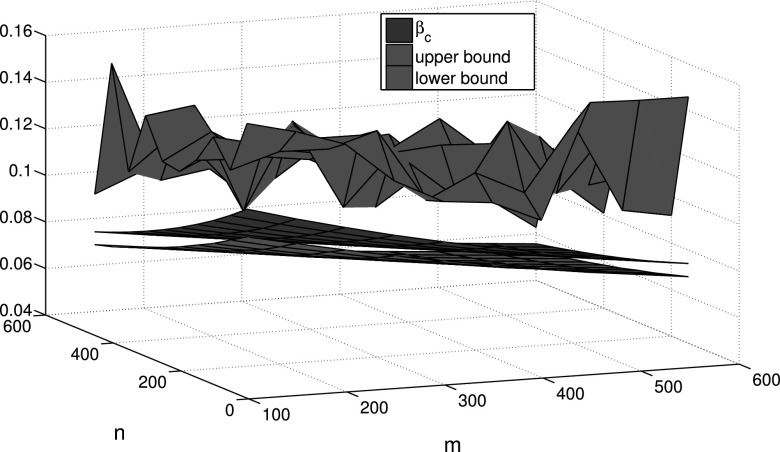
Comparisons between the epidemic threshold *β_c_* and upper-lower bound estimation under different community sizes *m* and *n*.

**FIG. 6.**
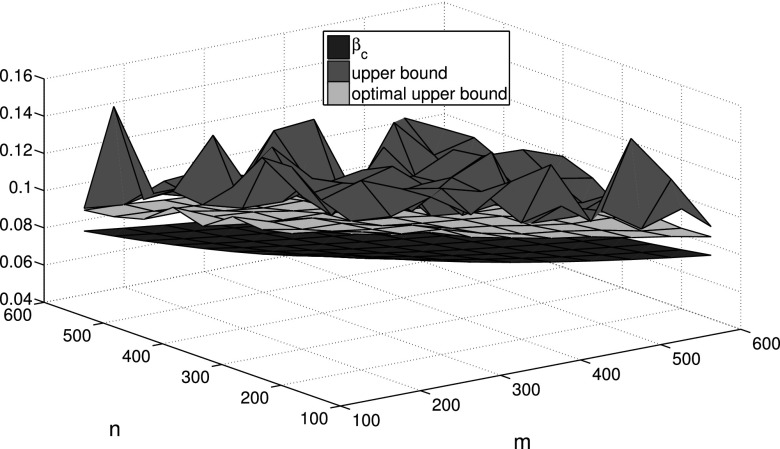
Comparisons between the epidemic threshold *β_c_*, upper bound, and optimal upper bound estimation under different community sizes *m* and *n*.

Finally, we take into account the community structure within an epidemic network with uniform stochastic connections. Suppose that the deterministic network *G* has two communities with sizes *m* and *n*, which both have WS small-world network structure. By choosing all pairs of nodes from the different communities, each outer connection is randomly generated with probability *p*. In this particular example, we choose *p* = 0.01 and a uniform stochastic probability *α* = 0.01. Under different community sizes *m* and *n*, Fig. 5 gives some comparisons between the epidemic threshold and upper-lower bound estimation in Theorem 4. In this figure, there exist a big gap between the epidemic threshold and upper bound. In order to decrease this gap, we can utilize the optimal upper bound estimation in Corollary 3. For this purpose, we give a realization in Fig. 6 which shows smaller gap than Fig. 5.

These simulation examples illustrate the correctness of theoretical results in Secs. III–V. Hence, in real epidemic networks, we can use only the information concerning deterministic connections (while ignoring stochastic connections) to estimate the epidemic thresholds.

## VII. CONCLUSIONS

In this paper, we focus on the estimation of the epidemic threshold on networks with deterministic and stochastic connections. First, we have constructed several epidemic models with some general properties, including nonuniform stochastic connections, local protection awareness of individuals, and community structure. Second, by using the spectral analysis on these networks, we have obtained some inequality estimates of their epidemic thresholds. The results show that these inequalities are only dependent on the topological structure of deterministic connections and the stochastic connection probabilities. In other words, one can use the information of deterministic connections, but not necessarily from all connections, to estimate the epidemic threshold. This work provides a feasible method for us to estimate the epidemic thresholds in real epidemic networks, when complete description of the stochastic nature of the epidemic may be difficult to obtain.

To further understand the epidemic dynamics in real complex networks there are, of course, topics which need to be resolved in the future. These include the network with nonuniform stochastic connections and community structure, the network with multi-community structure, among many others. Another important problem is to develop more effective method to improve the estimation power.
